# miR-548d-3p inhibits osteosarcoma by downregulating *KRAS*

**DOI:** 10.18632/aging.102097

**Published:** 2019-07-21

**Authors:** Jianhua Chen, Chongnan Yan, Honghao Yu, Shihan Zhen, Quan Yuan

**Affiliations:** 1 Department of Orthopedics, Shengjing Hospital of China Medical University, Shenyang 110001, People’s Republic of China

**Keywords:** osteosarcoma, miR-548d-3p, KRAS, growth, migration

## Abstract

MicroRNAs (miRNAs) are known to be associated with certain cancers, including osteosarcoma. We examined osteosarcoma tissues and cell lines, and found that most expressed lower levels of miR-548d-3p than adjacent tissues and normal cell lines. *KRAS* was identified as a potential target gene of miR-548d-3p. In osteosarcoma cells, miR-548d-3p exerted tumor-suppressive effects by downregulating *KRAS*. Functional assays revealed that miR-548d-3p mimics dramatically reduced cell growth and migration *in vitro*. These results suggest that miR-548d-3p mimics could be applied for osteosarcoma treatment.

## INTRODUCTION

Osteosarcoma accounts for about 0.2% of malignant tumors in humans. Osteosarcoma is most common in persons 15–25 years old, and is more common in men than in women. The development and progression of osteosarcoma are complex processes involving many genes, so the underlying mechanisms remain unclear [1, 2].

MicroRNAs (miRNAs) are small noncoding RNAs that downregulate genes by binding to their 3' untranslated regions (UTRs). Due to their ability to inhibit the expression of oncogenes and tumor suppressor genes, miRNAs are important contributors to the initiation and progression of tumor growth. The dysregulation of miRNAs has been linked to the proliferation, migration and prognosis of osteosarcoma [3, 4]. The binding of miR-101 to *ZEB2* prevents the proliferation and invasion of osteosarcoma cells [5]. MiR-144 inhibits the growth and migration of osteosarcoma cells by dually suppressing the RhoA/ROCK1 signaling pathway [6]. MiR-548 has become an attractive target for the treatment of cancers such as esophageal squamous cell carcinoma and prostate cancer, since its involvement in certain cancers has already been demonstrated [7, 8].

The *RAS* gene family (*HRAS*, *KRAS* and *NRAS*) is a group of early oncogenes that contribute to the development of a wide variety of tumors. Many studies have demonstrated that *KRAS* gene mutations are associated with osteosarcoma. Previous research in mice and humans has suggested that *RAS* gene products could be therapeutic targets in cancer, so targeted therapy for *KRAS* could be a new treatment possibility for osteosarcoma [9–11].

In the present study, we evaluated the expression of miR-548d-3p and *KRAS* in osteosarcoma. We examined whether miR-548d-3p could suppress the growth and migration of osteosarcoma cells by downregulating *KRAS*, thus providing a new target for the treatment of osteosarcoma.

## RESULTS

### The expression of miR-548d-3p and *KRAS* in osteosarcoma

By performing a microarray analysis, we identified seven miRNAs that were abnormally expressed in 3 osteosarcoma samples compared with adjacent tissues. Among these, the difference in miR-548d-3p expression was the most significant (Table 1).

**Table 1:** Primers used.

**Name**	**Forward primer (5'->3')**	**Reverse primer (5'->3')**
*KRAS*	GACTCTGAAGATGTACCTATGGTCCTA	CATCATCAACACCCTGTCTTGTC
*GAPDH*	AGAAGGCTGGGGCTCATTTG	AGGGGCCATCCACAGTCTTC
*miR-548d-3p*	CTCCAGCAAAAACCACAGTTTC	CTCAACGCAAAGAAACTGTGG
*U6*	CTCGCTTCGGCAGCACA	AACGCTTCACGAATTTGCGT

**Figure 1:**
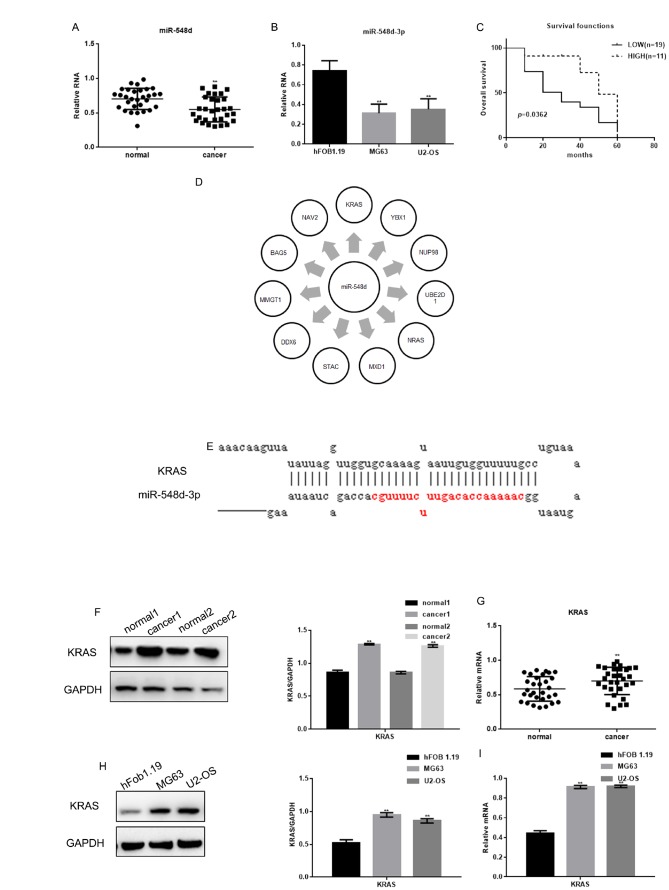
**The expression of miR-548d-3p and *KRAS* in osteosarcoma.** (**A**) The relative miR-548d-3p levels in 30 pairs of osteosarcoma tissues and adjacent normal tissues were detected by real-time PCR. Patients with lower miR-548d expression in their tumors than in mean of the normal. The results represent the mean±SD. **P < 0.05 vs. adjacent normal tissues. (**B**) Relative miR-548d-3p expression in cell lines (hFOB 1.19, MG63 and U2-OS). MG63 and U2-OS cells with lower miR-548d expression than hFOB 1.19 cells. The results represent the mean±SD of three independent experiments. **P < 0.05 vs. hFOB 1.19 cells. (**C**) The correlation between miR-548d-3p expression and patient survival. (**D**) Software analysis with miRDB revealed miR-548d-3p binding sites in a variety of genes. (**E**) The miRDB tool predicted that miR-548d-3p may bind to *KRAS*. (**F**) The relative KRAS levels in two pairs of osteosarcoma tissues and adjacent normal tissues were detected by Western blotting. The results represent the mean±SD. **P < 0.05 vs. adjacent normal tissues. (**G**) The relative *KRAS* levels in 30 pairs of osteosarcoma tissues and adjacent normal tissues were detected by Western blotting and real-time PCR. The results represent the mean±SD. **P < 0.05 vs. adjacent normal tissues. (**H**, **I**) The relative *KRAS* levels in cell lines (hFOB 1.19, MG63 and U2-OS) were detected by Western blotting and real-time PCR. The results represent the mean±SD of three independent experiments. **P < 0.05 vs. hFOB 1.19 cells.

The expression of miR-548d-3p in osteosarcoma tissues was found to correlate with the tumor grading (Table 2). The expression of miR-548d-3p was detected by real-time PCR in osteosarcoma tissues and human cell lines (hFOB 1.19, MG63 and U2-OS cells). As expected, miR-548d-3p expression was significantly lower in osteosarcoma tissues and cells (MG63 and U2-OS cells) than in adjacent tissues or hFOB 1.19 cells (Figure 1A and 1B). Patients with lower miR-548d expression in their tumors than in mean of the normal. Patients with lower miR-548d-3p expression in their tumors were named as the low expression group. The patients were followed up for 60 months, and survival was found to be worse in those with lower miR-548d-3p expression (Figure 1C). The median survival time was 30 months in the low miR-548d-3p expression group and 50 months in the high expression group.

**Table 2:** Differentially expressed miRNAs in osteosarcoma.

**miRNA**	**Fold change**	**Trend**
miR-548d-3p	54.34	down
miR-135a	46.41	down
miR-519b-5p	40.50	up
miR-365	32.71	down
miR-190	21.38	up
miR-299	17.72	up
miR-217	9.18	down

We then performed an analysis on the miRDB website (http://www.mirdb.org/), and identified miR-548d-3p binding sites in a variety of genes (Figure 1D). The miRDB tool indicated that miR-548d-3p could bind to the 3' UTR of *KRAS* (Figure 1E). We therefore assessed *KRAS* expression in osteosarcoma tissues and cells. Both the protein and mRNA levels of *KRAS* were significantly higher in osteosarcoma tissues and osteosarcoma cells than in adjacent tissues and hFOB 1.19 cells (Figure 1F–1I).

### MiR-548d-3p inhibits *KRAS* in osteosarcoma cells

**Figure 2:**
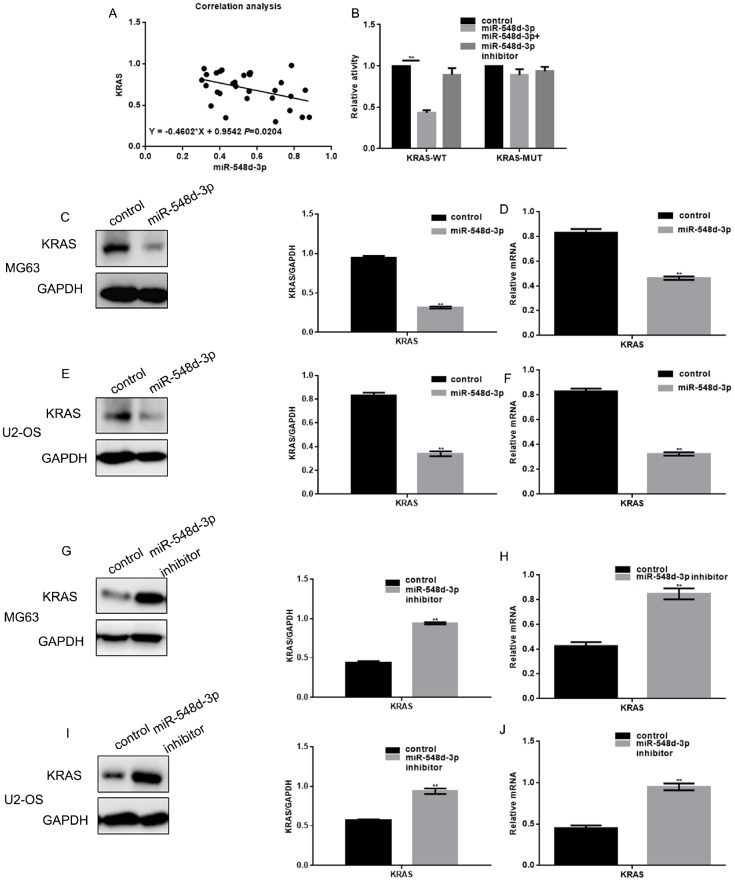
**MiR-548d-3p inhibits *KRAS* in osteosarcoma cells.** (**A**) Correlation analysis between miR-548d-3p and *KRAS* expression. The correlation formula was y=-0.4602x+0.9542. (**B**) A luciferase reporter gene assay confirmed that miR-548d-3p could bind to *KRAS*. The results represent the mean±SD of three independent experiments. **P < 0.05 vs. control. (**C–J**) The protein and mRNA levels of *KRAS* were measured in MG63 and U2-OS cells by Western blotting and real-time PCR, respectively. The results represent the mean±SD of three independent experiments. **P < 0.05 vs. control.

A Pearson correlation analysis demonstrated that miR-548d-3p expression correlated negatively with *KRAS* expression in osteosarcoma tissues (Figure 2A). A luciferase reporter assay was used to determine whether *KRAS* was a potential target gene of miR-548d-3p (Figure 2B). When hFOB 1.19 cells were co-transfected with KRAS-WT vectors and miR-548d-3p mimics, luciferase activity was reduced compared with cells co-transfected with KRAS-WT vectors and control. On the other hand, the luciferase activity was unchanged when co-expression studies were performed with KRAS-MUT vectors or miR-548d-3p inhibitors.

Further, when cells were transfected with miR-548d-3p mimics, *KRAS* expression was downregulated at both the mRNA and protein levels. Conversely, when cells were transfected with a miR-548d-3p inhibitor, *KRAS* was upregulated (Figure 2C–2J).

### MiR-548d-3p inhibits the growth and migration of osteosarcoma cells

**Figure 3:**
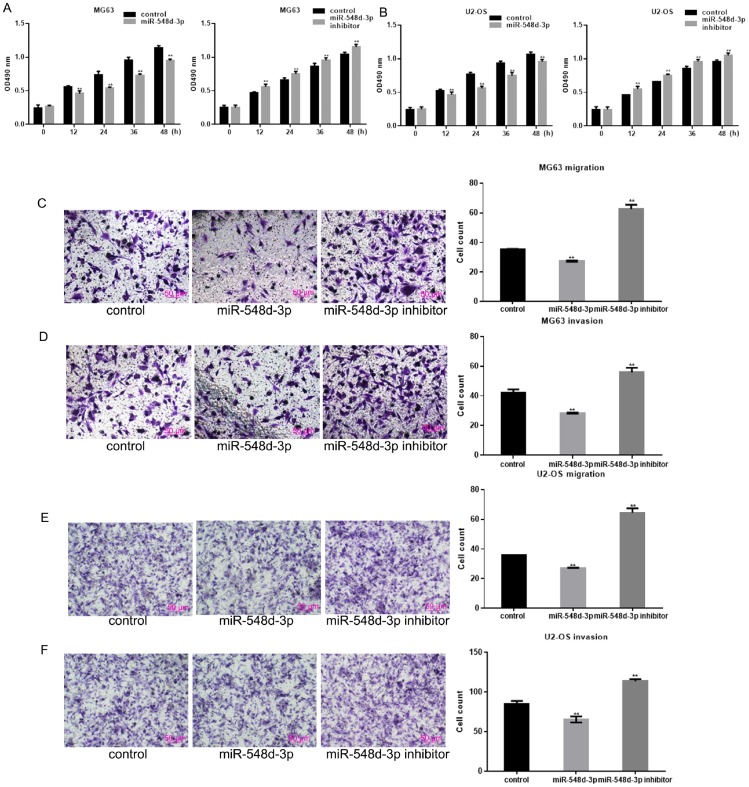
**MiR-548d-3p inhibits the growth and migration of osteosarcoma cells.** (**A**, **B**) MG63 and U2-OS cell growth was measured by an MTT assay after 24 h of transfection. The results represent the mean±SD of three independent experiments. **P < 0.05 vs. control. (**C**–**F**) The migration and invasion capacities of MG63 and U2-OS cells were measured by a Transwell assay after the cells were transfected with miR-548d-3p mimics, inhibitors or control miRNA for 24 h. The results represent the mean±SD of three independent experiments. **P < 0.05 vs. control.

An MTT assay was used to examine the effects of miR-548d-3p on cell growth. Overexpression of miR-548d-3p significantly inhibited the growth of both MG63 and U2-OS cells (Figure 3A). In contrast, inhibition of miR-548d-3p promoted cell growth (Figure 3B). Transwell assays revealed that miR-548d-3p significantly suppressed the migration and invasion potential of osteosarcoma cells (Figure 3C–3F).

### MiR-548d-3p inhibits the growth and migration of osteosarcoma cells by downregulating *KRAS*

**Figure 4:**
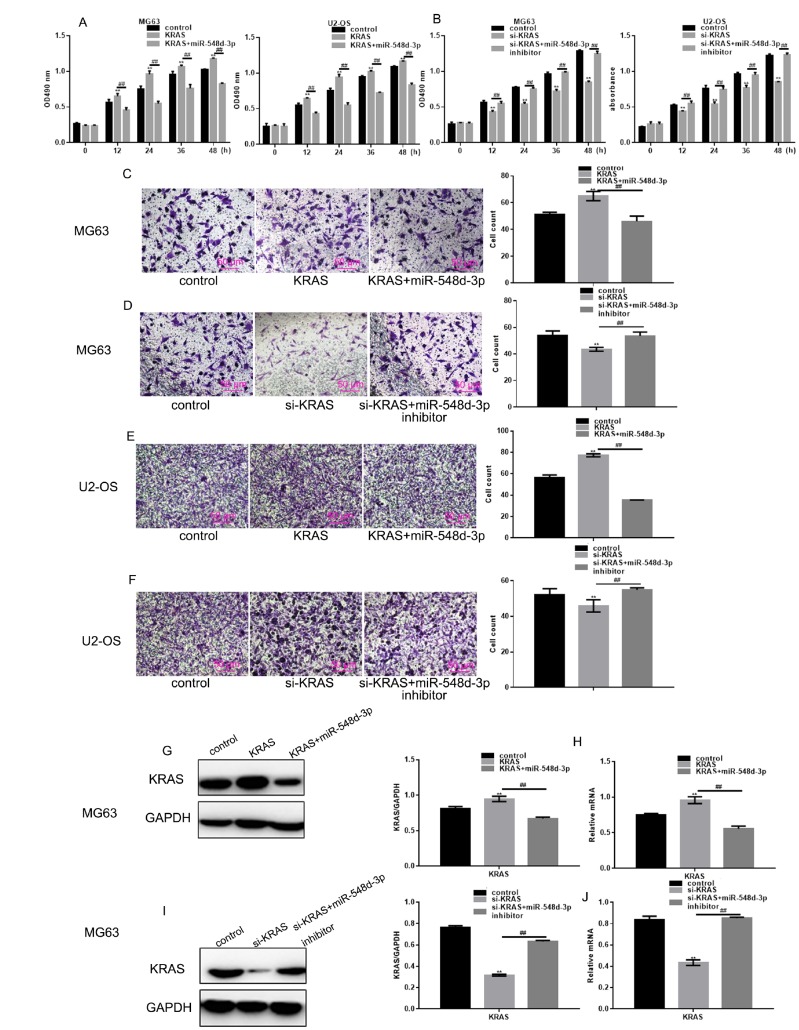
**MiR-548d-3p inhibits the growth and migration of osteosarcoma cells by downregulating *KRAS*.** (**A**) After transfection with *KRAS* overexpression vectors, cells were transfected with miR-548d-3p mimics or control miRNA. Cell growth was measured with an MTT assay after 24 h of transfection. The results represent the mean±SD of three independent experiments. **P < 0.05 vs. control, ##*P* < 0.05 vs. *KRAS* overexpression group. (**B**) After transfection with si-*KRAS*, cells were transfected with miR-548d-3p inhibitors or control miRNA. Cell growth was measured by an MTT assay after 24 h of transfection. The results represent the mean±SD of three independent experiments. **P < 0.05 vs. control, ##*P* < 0.05 vs. *KRAS* group or si-*KRAS* group. (**C**–**F**) Cell migration was measured with a Transwell assay after 24 h of transfection. The results represent the mean±SD of three independent experiments. **P < 0.05 vs. control, ##*P* < 0.05 vs. *KRAS* group or si-*KRAS* group. (**G**–**N**) The protein and mRNA levels of *KRAS* were measured in cells by Western blotting and real-time PCR, respectively. The results represent the mean±SD of three independent experiments. **P < 0.05 vs. control, ##*P* < 0.05 vs. *KRAS* group or si-*KRAS* group.

*KRAS*-overexpressing or si-*KRAS*-expressing cells were generated, and the cells were transfected with miR-548d-3p mimics, miR-548d-3p inhibitors or control miRNA. The effects of miR-548d-3p on cell growth and migration were then detected by MTT and Transwell assays. *KRAS* overexpression induced cell growth and migration, but the overexpression of miR-548d-3p weakened these effects (Figure 4A, 4C, 4E). On the other hand, transfection with si-*KRAS* inhibited cell growth and migration, whereas co-transfection with miR-548d-3p inhibitors restored these functions (Figure 4B, 4D, 4F). Western blotting and real-time PCR analysis demonstrated that the overexpression of miR-548d-3p suppressed the overexpression of *KRAS*, while the inhibition of miR-548d-3p re-induced the expression of silenced *KRAS* (Figure 4G–4N).

### MiR-548d-3p inhibits osteosarcoma

**Figure 5:**
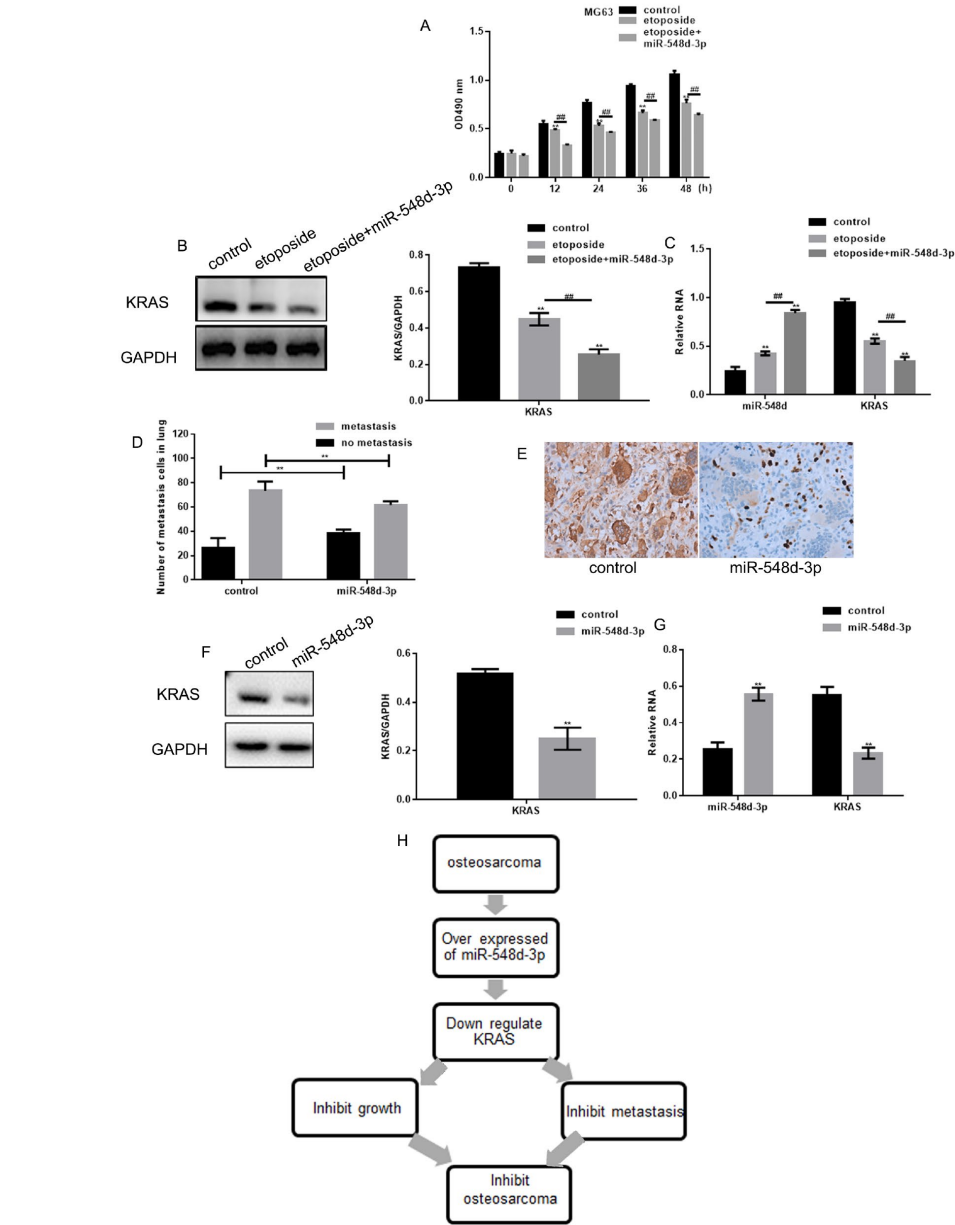
**MiR-548d-3p inhibits osteosarcoma.** (**A**) MG63 cell growth was measured with an MTT assay after treatment with control miRNA, 10 μM etoposide combined with control miRNA, or 10 μM etoposide combined with miR-548d-3p mimics. The results represent the mean±SD of three independent experiments. **P < 0.05 vs. control, ##*P* < 0.05 vs. etoposide. (**B**, **C**) MG63 cells were treated with control miRNA, 10 μM etoposide combined with control miRNA, or 10 μM etoposide combined with miR-548d-3p mimics. The protein and mRNA levels of *KRAS* were then measured by Western blotting and real-time PCR, respectively. **P < 0.05 vs. control, ##*P* < 0.05 vs. etoposide. The results represent the mean±SD of three independent experiments. (**D**) The migration of osteosarcoma cells to the lungs in mice treated with miR-548d-3p agomirs or control miRNA. The results represent the mean±SD. **P < 0.05 vs. control. (**E**) Immunohistochemical staining revealed the expression of KRAS in osteosarcoma in the control group and the miR-548d-3p overexpression group. (**F**, **G**) The protein and mRNA levels of *KRAS* were measured in MG63 cells by Western blotting and real-time PCR, respectively. The results represent the mean±SD of three independent experiments. **P < 0.05 vs. control. (**H**) The mechanism proposed in this article.

The effects of miR-548d-3p were then examined in cells treated with etoposide, a known anti-cancer drug. Etoposide treatment significantly downregulated the growth of MG63 cells, and mimics of miR-548d-3p further inhibited the growth of these cells (Figure 5A). Additionally, miR-548d-3p enhanced the etoposide-induced downregulation of *KRAS* (Figure 5B and 5C).

Osteosarcoma tumor growth was then induced in mice by the injection of MG63 cells. Treatment with miR-548d-3p agomirs inhibited the lung migration of osteosarcoma cells in these mice (Figure 5D). Moreover, miR-548d-3p treatment downregulated *KRAS* expression *in vivo* osteosarcoma tissues (Figure 5E–5G). Thus, we speculate that miR-548d-3p may inhibit osteosarcoma by downregulating *KRAS* (Figure 5H).

## DISCUSSION

Despite improvements in surgery and adjuvant chemotherapy, the clinical prognosis of osteosarcoma is still very poor [12, 13]. The reasons for a poor prognosis are typically pulmonary migration and drug resistance [14].

Various miRNAs have been found to contribute to the initiation and progression of osteosarcoma [15]. A continued search for such miRNAs is needed to provide new targets and treatment strategies for this disease. The miR-548 family is involved in the pathogenesis of several cancers. For instance, miR-548 was found to inhibit the growth of breast cancer cells by downregulating *ECHS1* [16], and the downregulation of miR-548an was reported to promote pancreatic tumorigenesis [17]. However, the involvement of miR-548 in osteosarcoma has not previously been elucidated. Thus, we explored the expression and function of miR-548d-3p in clinical specimens and osteosarcoma cells.

The present studies have showed that miR-548 can be considered as potential targets in prostate cancer and breast cancer therapy [7, 16, 18]. In our study, miR-548d-3p was found to be downregulated in osteosarcoma. Patients with high miR-548d-3p expression survived longer than patients with low miR-548d-3p expression. Researchers showed that miR-548-3p could regulat the expression of ECHS1 and NRIP1 [16, 18]. We searched for potential target genes of miR-548d-3p, and identified *KRAS.* *RAS* is involved in the development of a variety of tumors, and *KRAS* has been used as a prognostic marker in clinical oncology [9]. In our osteosarcoma samples, *KRAS* expression correlated negatively with miR-548d-3p expression. In addition, the upregulation of miR-548d-3p significantly inhibited *KRAS* expression, while the downregulation of miR-548d-3p significantly increased *KRAS* expression. Further studies indicated that miR-548d-3p could bind directly to *KRAS*. Study has shown that overexpression of miR-548-3p can inhibit the proliferation of breast cancer cells [16]. MiR-548-3p has also been shown to play an important role in regulating the migration and invasion of esophageal cancer cells [7]. Our research showed that treatment with miR-548d-3p mimics significantly inhibited the growth and migration of osteosarcoma cells. Through a series of experiments, we demonstrated that the overexpression of miR-548d-3p weakened the effects of KRAS on cell growth and migration, while the inhibition of miR-548d-3p reversed the effects of *KRAS* silencing. Our *in vivo* experiments confirmed that the overexpression of miR-548d-3p could suppress the migration of osteosarcoma cells, possibly by inhibiting *KRAS*.

We also found that miR-548d-3p mimics enhanced the sensitivity of cancer cells to etoposide, an approved anti-cancer drug, at least to a certain extent. Various drugs have been shown to inhibit the development of osteosarcoma by inhibiting RAS [19]. We suggest that miR-548d-3p mimics may increase the inhibitory effects of RAS inhibitors in the same way that they enhance the effects of etoposide.

In summary, although further *in vivo* tests are needed to confirm our results, our data have illustrated that miR-548d-3p can inhibit the growth and migration of osteosarcoma cells by downregulating *KRAS*.

## MATERIALS AND METHODS

### Samples

Tissues were obtained from 30 patients undergoing surgery at Shengjing Hospital. The average age of the patients was 20.37 years (ranging from 11 to 39 years). The original histopathologic report was obtained for each patient, and the diagnosis of osteosarcoma was confirmed. All clinical samples were collected with written informed consent from the patients, and the study was approved by the Ethics Committee at the Academic Medical Center of China Medical University.

### MiRNA microarray analysis

In total, three paired tumor and adjacent normal tissue specimens were randomly selected. Total RNA was isolated with TRIzol reagent (Invitrogen, Carlsbad, CA, USA) and an miRNeasy Mini kit (Qiagen, Valencia, CA, USA) according to the manufacturers’ instructions. Samples with an RNA integrity number >8 were processed for hybridization. Total RNA was labeled with a miRCURY™ Array Power labeling kit (Exiqon, Copenhagen, Denmark) and hybridized to the miRNA microarray with a miRCURY LNA™ microRNA Array kit (Exiqon). Images were then obtained with a GenePix 4000B microarray scanner and imported with the associated software (Axon Instruments, Sunnyvale, CA, USA). SpotData Pro software was used for data analysis. Hierarchical clustering was performed with Data Matching Software.

### Cell culture

MG63, hFOB 1.19 and U2-OS cells were purchased from the China Center for Type Culture Collection (CCTCC). Cells were cultivated in Dulbecco’s modified Eagle’s medium (Invitrogen) supplemented with 10% fetal bovine serum (FBS, Invitrogen) and incubated with 5% CO_2_ at 37°C.

### Transfection

MiR-548d-3p mimics (CAAAAACCACAGTTTCTTTTGC), miR-548d-3p inhibitors (GCAAAAGAAACTGTGGTTTTTG) and control miRNA (TTCTCCGAACGTGTCACGT), as well as *KRAS* siRNA (‘si-*KRAS*’, CGAGTGGTTGTACGATGCATTGGTT) and control siRNA (CGTACGCGGAATACTTCGA), were provided by RiboBio (Guangzhou, China), and were used for transfection at a final concentration of 50 nmol/L. *KRAS* overexpression (pcDNA-KRAS) and empty (pcDNA-3.1) vectors were purchased from GeneChem, and 3.2 μg of the vectors were used for transfection in six-well plates. Lipofectamine 2000 (Invitrogen) was used for cell transfection in accordance with the manufacturer’s protocols. The miRNAs or expression vectors were mixed with the medium and Lipofectamine 2000 for 5 min and then added to the cells. Protein and total RNA were extracted 24 h after transfection.

### MTT assays

Cell growth was estimated by the MTT assay. Cells were seeded into 96-well plates at a density of 1,000 cells per well and incubated at 37°C. After 24 h, the cells were transfected or treated as indicated. At the end of the incubation, 10 μL of MTT solution (5 mg/mL) was added to each well, and the media were replaced with 200 μL dimethyl sulfoxide after incubation for 4 h. The optical density at 490 nm was measured on a microplate reader (Bio-Rad, Shanghai, China). The experiments were repeated independently three times.

### Migration assay

For the migration and invasion assays, 1×10^5^ cells were seeded onto the upper part of a Transwell chamber (Corning-Costar, USA) with or without a gelatin-coated polycarbonate membrane filter (pore size: 8 μm). Cells resuspended in FBS-free medium were added to the upper well, and the lower well was filled with 10% FBS. The cells were cultivated at 37°C for 24 h. Invasive cells on the bottom of the membrane were fixed and stained. An inverted microscope was used to view the cells, and five random fields per chamber were counted. The experiments were repeated independently three times.

### Luciferase assay

The entire 3′-UTR of human *KRAS* was amplified via PCR with human genomic DNA as a template. Then, the PCR products were inserted into the pMIR-REPORT plasmid (Ambion, USA), and successful insertion was confirmed by DNA sequencing. For the assessment of binding specificity, the *KRAS* sequence known to bind to the seed region of miR-548d-3p was mutated (from GCAAAAGTA to CGTTTTCAT), and the mutant *KRAS* 3′-UTR was inserted into an equivalent luciferase reporter.

For the luciferase reporter assays, hFOB 1.19 cells were seeded into 24-well plates. The cells were co-transfected with 1 μg of the firefly luciferase reporter plasmid, 1 μg of the β-galactosidase expression plasmid (Ambion) and equal amounts (100 pmol) of miR-548d-3p mimics, inhibitors or control miRNA by means of Lipofectamine 2000 (Invitrogen). The β-galactosidase plasmid was used as a transfection efficiency control. After the cells were incubated for 24 h, luciferase activity was measured with a Dual-Luciferase Reporter Assay System (Promega, Beijing, China). The experiments were repeated independently three times.

### Real-time PCR

Total RNA was extracted from cell lines with TRIzol (Invitrogen) according to the manufacturer’s protocol. Then, 2 μg of total RNA was reverse transcribed into first-strand cDNA with a reverse transcription reagent kit (TaKaRa, Japan) according to the manufacturer’s protocol. Quantitative PCR was performed with a SYBR Green real-time PCR kit (TaKaRa, Japan). The primers listed in Table 3 were synthesized by Shenggong (Shanghai, China). Relative miRNA and mRNA levels were determined by the 2^−ΔΔCt^ method, and were normalized to *U6* or *GAPDH* levels. The experiments were repeated independently three times.

**Table 3:** The relationship between miR-548d-3p expression and osteosarcoma.

**Variables**	**Description**	**No. of patients**	**miR-548d-3p expression**		**χ^2^**	***P* value**
			**Low**	**High**		
Gender	Male	17	11	6	0.032	0.858
	Female	13	8	5		
Age (years)	<20	18	10	8	1.17	0.279
	≥20	12	9	3		
Family history	No	23	14	9	0.258	0.612
	Yes	7	5	2		
TNM grade	I	13	5	8	6.415	0.040*
	IIA	15	12	3		
	IIB	2	2	0		

### Western blot analysis

Total protein was extracted from cells with a lysis buffer (Beyotime, China) containing proteinase inhibitors. Equal amounts of protein (30 μg) were boiled at 100°C for 5 min, separated on 10% polyacrylamide gels and transferred to polyvinylidene difluoride membranes (Millipore, Bedford, MA, USA). The membranes were probed with a specific antibody against KRAS (sc-30, 1:1000; Santa Cruz, USA), and were stripped and re-probed with an antibody against GAPDH (SC-32233, 1:1000; Santa Cruz, USA) to verify equal loading. Protein levels were quantified with Image J software (National Institutes of Health). The experiments were repeated independently three times.

### Nude mouse experiment

Twelve five- to six-week-old female nude BALB/c mice (Vital River Laboratory Animal Technology Co. Ltd., China) were each injected with 2×10^6^ MG63 cells in a 200-μL cell suspension, and the metastatic ability of the cells was investigated. The mice were divided into two groups (n=6/group): the miR-548d-3p agomir group (CGTTTTCTTTGACACCAAAAAC) and the negative control group (TTCTCCGAACGTGTCACGT). On day 21 following the injection, osteosarcoma tumor samples were collected for immunohistochemistry, and the lungs were removed and evaluated for visible tumor nodules.

All experimental procedures involving animals were conducted in accordance with the Guide for the Care and Use of Laboratory Animals (NIH publication no. 80-23, revised 1996) and the institutional ethical guidelines for animal experiments.

### Immunohistochemistry

Immunohistochemistry was performed as described previously [20]. The immunohistochemical results were judged by the histological score (HSCORE) [21].

### Statistical analysis

All experiments were repeated independently three times, and the results are shown as the mean and standard deviation (SD). Kaplan-Meier survival analysis with a log-rank test was used to analyze the overall survival of osteosarcoma patients. Pearson correlation analysis was used to evaluate the correlation between *KRAS* and miR-548d-3p expression. Student's t test (two-tailed) was used to evaluate the statistical significance of differences between two groups. Statistical analyses were performed with GraphPad Prism software (GraphPad, Inc.). Statistical significance was defined as *P* < 0.05.

### Compliance with ethical standards

Patients provided written consent and approval for the use of clinical materials for research purposes at Shengjing Hospital, in accordance with institutional regulations.
